# Event and Comment

**Published:** 1910-11-15

**Authors:** 


### DENTAL REGISTER.

**Vol. LXIV. November 15, 1910. No. 11**

### EVENT AND COMMENT.

PROFESSIONAL IDEALS. We are often tempted to pay small attention when anyone presents the more sentimental aspect of professional work for our consideration, we even may consider it tedious and unprofitable. This is liable to be the attitude of the inexperienced or the narrow and selfishly practical man. With the experience of years of activity in the broader fields of practice, matured by careful thinking, we inevitably come to the viewpoint of the philosopher who is for ever calling our attention to the fact that no great work is the result of mere activity. We must have an object that appeals to all that is noble and good in us, and this we call an ideal, because it must first be worked out through an enlightened intelligence. As a profession we have in a degree ignored ideals, because there seemed to be so much of the practical that demanded our immediate efforts, that we have remained unconscious of the fact that time would be conserved by a more deliberate consideration of our problem. In the past we have, to put it tersely, acted only on the *idea* that our duty was merely to relieve suffering and restore lost function of the teeth from the consequence of disease, and that loss of the teeth from caries and peridentitis was inevitable and that our whole duty was to retard and repair. Can we not see in the more recent professional activities a tendency to break away from this rather narrow view, into a broader conception of our calling? If the present general discussion of the subject of oral hygiene means anything, it is that the profession has caught the inspiration of an *ideal* that will give us a higher place in the great humanitarian work of the world. The utilization of our accumulated skill with so noble an ideal as a goal should make us great.

ORAL PROPHYLAXIS. At the next meeting of the Ohio State Dental Society the entire programme, except the clinical department, will be devoted to a consideration of the various aspects of those diseases which have to do with the loss of the teeth, and to preventive and rescue treatment. It may seem to some that this is an unwarrented use of the time of so great a meeting, because there are other subjects that equally need discussion. We believe, however, that the importance of the subject and the widespread interest in it at this time, because of the fact that it is fundamental to the higher conception of our duty as a profession, are sufficient to justify the programme committee in confining its literary work on this subject. The concentration of thought of so live and intelligent a body of workers on a subject which is so much talked about and worked over to-day, ought to result in greater intelligence, and give an impetus to preventive dentistry that will mean much for the future of the profession, not only in Ohio, but wherever its men may carry these ideals and the result of their deliberations. We anticipate a rare privilege in attending this meeting, and believe that the concentration of experience will result, not only in erecting new ideals, but in crystalizing methods of hygienic and therapeutic treatment of inestimable value.

